# Supplementary material for: The Optimization and Biological Significance of a 29-Host-Immune-mRNA Panel for the Diagnosis of Acute Infections and Sepsis
Source: J Pers Med. 2021 Jul 28;11(8):735. doi: 10.3390/jpm11080735 (PMC8402342; doi:10.3390/jpm11080735)
Supplement: Supplementary file 1 [file jpm-11-00735-s001.zip › jpm-1265325-supplementary.pdf]

Supplemental Material for:
The Optimization and Biological Significance of a 29-Host-Immune-mRNA Panel for the Diagnosis of Acute Infections and Sepsis

Table of Contents

Supplemental Methods

1. *Marker Downselection Rational and Methods*
2. *Databases Used for Functional Annotation and Pathway Analysis*
   2.1 *Gene-Centric Knowledge Base:* ***GeneCards***
   2.2 *A Hierarchically Structured Knowledge System****: Gene Ontology***
   2.3 *Curated Biological Pathway Database:* ***KEGG Pathway***
   2.4 *Curated Biological Reaction Database:* ***Reactome***
3. *Systems, Platforms, and Tools Used for Gene Set and Pathway Analysis*
   3.1 *IPA for Gene Network Construction and Upstream Regulator Analysis.*
   3.2 *Tools for Gene Set Enrichment Analysis*

References for Supplemental Methods and Results

Supplemental Tables
Supplemental Table 1. List of initial 29 biomarkers for InSep.
Supplemental Table 2. List of 64 potential alternative biomarkers for InSep diagnostic test.
Supplemental Table 3. Pool of 48 markers for machine learning marker selection.
Supplemental Table 4. The list of markers produced by Phase I marker swap.

Supplemental Methods

1. *Marker Downselection Rational and Methods:*

We used logistic regression (LOGR) in Phase I due to competitive performance on our datasets, and low computational complexity (fast training) of LOGR. We reasoned that the initial set of genes will comprise genes with relatively strong signal, and therefore detectable by any competitive machine learning algorithm. LOGR was judged to offer best trade-off between accuracy and complexity.

We further reasoned that tuning the set of markers to the target size of 29 would require using most accurate classifier because the signal from the additional markers is gradually weakening. To that end, Phase II used forward selection with MLP classifier, which has to date yielded most accurate models for classification of infections using host response data, and therefore was most likely to uncover the additional informative markers.

Phase I used the following variant of the forward-selection algorithm:

- Input: empty marker set M and full set of candidate markers F
- repeat for remaining markers (i.e., markers in F \ M)
    - add one marker
    - repeat for 100 logistic regression hyperconfigurations
        - repeat over 100 splits of 5-fold random CV
            - for each fold
                - train LOGR on the training set
                - compute probabilities for the validation set
            - pool validation set probabilities, calculate AUC
        - average AUC over 100 splits
    - calculate 95-th percentile of AUCs (over 100 hyperconfigurations)
    - stop if no improvement to AUC is achieved by adding new markers
    - add marker with best 95-th percentile of AUCs to M
- Output: minimal list M of markers which maximize AUC

Phase II also involved expert input because the weaker signal of the final 10 markers necessitated evaluation of multiple target metrics. Simultaneous assessment of multiple metrics with trade-offs in mind is not amenable to automation using generic computer optimization algorithms because they require a single loss (criterion) function.

Phase II used the following variant of the forward-selection algorithm, with human input:

- Input: marker set M and full set of candidate markers F
- repeat for remaining markers (i.e., markers in R = F \ M)
    - for each marker in R
        - add one marker to the training and validation sets.
        - select best MLP model using training set and cross-validation
        - apply the model to the validation set
        - record training (cross-validation) AUC and validation AUC. These statistics are a function of the marker
    - select best marker in R (human input) and move it to M
    - stop if M has 29 markers
- Output: minimal list of markers which achieve best tradeoff of diagnostic performance and robustness

2. *Databases/Systems Used for Functional Annotation and Pathway Analysis*

2.1 *Gene-Centric Knowledge Base:* ***GeneCards***

GeneCards[1], one of the leading resources on gene information, is a searchable, integrative database that provides comprehensive, user-friendly information on all annotated and predicted human genes. The system automatically integrates gene-centric data from ~150 web sources concerning genes and their products including genomic, transcriptomic, proteomic, genetic, clinical, and functional information. We primarily used GeneCards to acquire information about genes of interest and use other resources if further investigations are warranted.

2.2 *A Hierarchically Structured Knowledge System**: Gene Ontology***

Gene Ontology (**GO**)[2] is a vocabulary-controlled and hierarchically structured system designed to capture various function-related attributes for genes and gene products, as a formal representation of a body of knowledge within a given domain. Its goal was to enable functional interpretation of experimental data, for example via enrichment analysis. GO includes three domains: biological process (BP), molecular function (MF), and cellular compartment (CC). For example, the gene product "cytochrome c" can be described by **MF** *oxidoreductase activity*, **BP** *oxidative phosphorylation*, and **CC** *mitochondrial matrix*. Aiming to represent the current state of knowledge in biology, GO, both in its classes and graphs, are dynamic with capability to periodically acquire new knowledge via various sources. However, the level of confidence in gene annotation in GO varies dramatically, depending on the evidence code in 6 general categories with various levels (experimental evidence, phylogenetic evidence, computational evidence, author statements, curatorial statements, and automatically generated annotations) that denote the type of evidence upon which the annotation is based. Currently, the system has 44,085 GO terms and nearly 8 million annotations for over 1.5 million gene products.

2.3 *Curated Biological Pathway Database:* ***KEGG Pathway***

KEGG Pathway[3] is a collected reference of manually drawn pathway maps representing our knowledge of the molecular interaction, reaction, and relation networks for 1) metabolism, 2) genetic information processing, 3) environmental information processing, 4) cellular processes, 5) organismal systems, 6) human diseases, and 7) drug development. Contents of KEGG pathways are being continuously updated as new findings are reported. To some extent, information in KEGG pathway is regarded as "textbook knowledge" with high confidence.

A.2.4 *Curated Biological Reaction Database:* ***Reactome***

Reactome[4] collects biological pathways in specific organisms with human biology of our interest as the largest collection. It is authored by expert biologists, in collaboration with Reactome editorial staff who are all PhD level biologists. Content is cross-referenced to many bioinformatics databases. The rationale behind Reactome is to visually represent biological pathways in full mechanistic detail, while making the source data available in a computationally accessible format. The core unit of the Reactome data model is the reaction. Entities (nucleic acids, proteins, complexes, and small molecules) participating in reactions form a network of biological interactions and are grouped into pathways. Examples of biological pathways in Reactome include signaling, innate and acquired immune function, transcriptional regulation, translation, apoptosis, and classical intermediary metabolism. The pathways represented in Reactome are species-specific, with each pathway step supported by literature citations that contain an experimental verification of the process represented. If no experimental verification using human reagents exists, pathways may contain steps manually inferred from non-human experimental details, but only if

an expert biologist, named as author of the pathway, and a second biologist, names as reviewer, agree that this is a valid inference to make.

3 Systems, Platforms, and Tools Used for Gene Set and Pathway Analysis

Amongst various systems, platforms, tools one can use for gene-level and geneset-level pathway analysis, we used the following in our work presented in this report.

3.1 *Ingenuity Pathway Analysis for Gene Network Construction and Upstream Regulator Analysis.*

Ingenuity Pathway Analysis (**IPA**)[5] allows searching and using information extracted from public databases and from full text article extraction. It categorizes findings based on an in-house curated ontology, covering entities (proteins and other molecules), relationships between the entities (inhibition, activation, co-expression, etc.) and functional information (pathways, biological process, disease, etc.). The platform allows building biological networks on the fly based on their captured knowledge base as well as computing enrichment from a user defined gene list based on various pathway contents and biological terms contents IPA has acquired.

Based on our gene list, we reconstructed knowledge-based networks including genes and phenotype as node. We used IPA upstream regulator analysis[6] to identify upstream regulators whose changes may likely cause the observed gene expression changes in our markers. Similarly, we can also perform analysis to identify downstream biological processes that are increased or decreased based on the markers of interest. We also used IPA bioprofiler to explore phenotype-gene relationship with bioprofiler.

3.2 *Tools for Gene Set Enrichment Analysis*

g:Profiler[7] is a public web server available for functional enrichment analysis, as known as over-representation analysis (ORA) or gene set enrichment analysis (GSEA). It can be used to test the significance of over-representation of any given list of genes over terms captured by various contents, such as MF, PB, and CC in GO, pathways in KEGG pathway, or reactions in Reactome. We chose only annotated genes in human genome as background when estimating p-values for significance of input genes in each term under the basic assumption of a hyper-geometric distribution. For multiple testing correction, we selected g:SCS method as it is a better method in comparison with the commonly used Bonferroni adjustment and Benjamini-Hochberg corrections method[8,9]. This approach is based on the idea that standard multiple testing corrections such as Bonferroni correction and Benjamini-Hochberg FDR are designed for multiple tests that are independent of each other. This is certainly not correct for the analysis, since GO consists of hierarchically related general and specific terms. The True Path Rule of GO states that genes associated with a given GO term are implicitly associated to all more general parents of this term. g:SCS threshold is a value pre-calculated for query list sizes up to 1000 genes. Given a fixed input query size, g:SCS analytically approximates a threshold t corresponding to the 5% upper quantile of randomly generated queries of that size. All actual p-values resulting from the query are transformed to corrected p-values by multiplying these to the ratio of the approximate threshold t and the initial experiment-wide threshold 0.05. The algorithm considers the set structure underlying gene sets annotated to terms of each organism, and should therefore give a tighter threshold to significant results. g:SCS thresholds perfectly agreed in simulations with randomly generated gene sets of fixed input query sizes.

References

1. GeneCard. *https://www.genecards.org/*.

2. Gene Ontology. *http://geneontology.org/*.

3. KEGG Pathway. *https://www.genome.jp/kegg/pathway.html*.

4. Reactome. *https://reactome.org/*.

5. QIAGEN Ingenuity Pathway Analysis (QIAGEN IPA). *https://digitalinsights.qiagen.com/products-overview/discovery-insights-portfolio/analysis-and-visualization/qiagen-ipa/*.

6. Krä Mer A, Green J, Pollard J, Tugendreich S. Systems biology Causal analysis approaches in Ingenuity Pathway Analysis. 2014;30(4):523-530. doi:10.1093/bioinformatics/btt703

7. g:Profiler. *https://biit.cs.ut.ee/gprofiler/gost*.

8. Ri Reimand J, Kull M, Peterson H, Hansen J, Vilo J. g:Profiler-a web-based toolset for functional profiling of gene lists from large-scale experiments. *Nucleic Acids Res*. 2007;35:193-200. doi:10.1093/nar/gkm226

9. Raudvere U, Kolberg L, Kuzmin I, et al. g:Profiler: a web server for functional enrichment analysis and conversions of gene lists (2019 update). *Nucleic Acids Res*. 2019;47:191-198. doi:10.1093/nar/gkz369

10. Sweeney TE, Shidham A, Wong HR, Khatri P. A comprehensive time-course-based multicohort analysis of sepsis and sterile inflammation reveals a robust diagnostic gene set. *Sci Transl Med*. 2015;7(287):1-16. doi:10.1126/scitranslmed.aaa5993

11. Sweeney TE, Wong HR, Khatri P. Robust classification of bacterial and viral infections via integrated host gene expression diagnostics. *Sci Transl Med*. 2016;8(346). doi:10.1126/scitranslmed.aaf7165

12. Sweeney TE, Perumal TM, Henao R, et al. A community approach to mortality prediction in sepsis via gene expression analysis. *Nat Commun*. 2018;9(1). doi:10.1038/s41467-018-03078-2

Supplemental Tables

Supplementary Table S1. List of initial 29 biomarkers for InSep. The list includes 1) 11 mRNAs diagnosing the presence or absence of an acute infection[10], 2) 7 mRNAs for distinguishing an infection between bacterial and viral[11], and 3) 12 mRNAs for determining the risk of 30-day mortality from sepsis[12]. Colored green genes are over-expressed, and red are under-expressed.

Infection vs SIRS/trauma	Fever	Severity
CEACAM1	IFI27 (viral)	DEFA4
ZDHHC19	JUP (viral)	CD163,
C9orf95	LAX1 (viral)	PER1
GNA15	HK3 (bacterial)	RGS1
BATF	TNIP1 (bacterial)	HIF1A
C3AR1	GPAA1 (bacterial)	SEPP1
KIAA1370	CTSB (bacterial)	C11orf74
TGFBI		CIT
MTCH1		LY86
RPGRIP1		TST
HLA-DPB1		OR52R1

Supplementary Table S2. List of 64 potential alternative biomarkers for InSep diagnostic test. Candidates are grouped by presumed compatibility with measurement by qRT-LAMP into four tiers. Tier 1 markers are predicted to be most amenable to qRT-LAMP analysis. Tier 1 and 2 genes are considered candidate pool except CD24 and SUCLG2 that failed gDNA screening.

Tier 1	Tier 2	Tier 3	
ARG1	CTLA4	CD97	HTRA1
CD24*	FURIN	IL16	KCNMB4
CTSL1	HAL	CEACAM4	KIF2C
DDX6	HLA-DMB	LAMP1	KPNA1
FCER1A	RAPGEF1	PLA2G7	PPL
GADD45A	SUCLG2*	PLAC8	PRC1
ICAM1	TMEM19	BTN2A2	RAB3GAP1
ISG15	ZBTB33	C1QA	RHBDF2
LCN2		CAMK4	SENP5
LTF		CRISP2	SLC12A7
OASL		CYCS	SLC25A22
OLFM4		DDIT4	STX1A
PDE4B		DSC1	TCF4
PLEKHO1		DTX2	TLR5
PSMB9		EBI3	TP53BP1

RELB		EPHB2	TWISTNB
S100A12		FRS2	YKT6
SAMSN1		HESX1	ZCCHC4
SMARCD3			ZWINT

Supplementary Table S3. Pool of 48 markers for machine learning marker selection.

ARG1	CTLA4	FURIN	HLA-DMB	KCNJ2	MTCH1	PSMB9	SMARCD3
BATF	CTSB	GADD45A	HLA-DPB1	KIAA1370	OASL	RAPGEF1	TGFBI
C3AR1	CTSL1	GNA15	ICAM1	LAX1	OLFM4	RELB	TMEM19
C9orf95	DDX6	HAL	IFI27	LCN2	PDE4B	RGS1	TNIP1
CD163	DEFA4	HIF1A	ISG15	LTF	PER1	S100A12	ZBTB33
CEACAM1	FCER1A	HK3	JUP	LY86	PLEKH01	SAMSN1	ZDHHC19

Supplementary Table S4. The list of markers produced by Phase I marker swap.

ARG1	CTSB	GADD45A	OASL	RAPGEF1
C9orf95	CTSL1	GNA15	OLFM4	TGFBI
CD163	DEFA4	HLA-DMB	PDE4B	ZDHHC19
CEACAM1	FURIN	IFI27	PER1	
